# Spatial variation and determinant factors of alcohol consumption in Ethiopia: Spatial and multilevel analysis of Ethiopian demographic and health survey

**DOI:** 10.1371/journal.pone.0309943

**Published:** 2025-01-08

**Authors:** Chala Daba, Sisay Abebe Debela, Kassahun Ayele Gasheya, Abel Endawkie, Mesfin Gebrehiwot

**Affiliations:** 1 Department of Environmental Health, College of Medicine and Health Sciences, Wollo University, Dessie, Ethiopia; 2 Department of Public Health, College of Health Science, Salale University, Fitche, Ethiopia; 3 Department of Occupational Health and Safety, College of Medicine and Health Sciences, Wollo University, Dessie, Ethiopia; 4 Department of Epidemiology and Biostatistics, School of Public Health, College of Medicine and Health Sciences, Wollo University, Dessie, Ethiopia; Debre Berhan University, ETHIOPIA

## Abstract

**Background:**

Alcohol consumption continues to be a public health problem in Ethiopia. Previous investigations have been conducted on alcohol consumption in Ethiopia; however, these investigations were limited to specific localities, which could not represent the existing alcohol consumption in different parts of Ethiopia. Besides, the spatial variation of alcohol consumption was not well investigated in the previous studies, which could hinder the implementation of effective intervention towards alcohol consumption. Hence, this study aimed to determine the spatial distribution and determinant factors of alcohol consumption in Ethiopia.

**Methods:**

Secondary data from the 2016 Ethiopian demographic health survey was used in this study. A total of 44,023 weight samples were included using a stratified two-stage cluster sampling technique. The spatial variation of alcohol consumption was analyzed using ArcGIS version 10.7.1. The statistical significance of alcohol consumption clusters were identified using Kuldorff’s SaTScan version 10.2. A multi-level analysis was also conducted to identify factors associated with alcohol consumption using STATA version 14.

**Results:**

In this study, 33.15% (95%CI: 32.5–33.8) of the study participants consumed alcohol with statistically significant spatial variation across regions of the country. Traditional religion (AOR = 13.7; 95%CI: 2.68–70.3), Regional variations (Amhara region—AOR = 3.56; 95%CI: 1.85–6.8, living in a low proportion of community literacy (AOR = 1.84; 95%CI: 1.1–3.18), cigarette smoking habit (AOR = 15.82; 95%CI: 4.31–58.1), and chewing *Khat* (AOR = 2.98; 95%CI: 1.22–7.27) were positively linked with alcohol consumption. Hot spot areas of alcohol consumption were found in Tigray, Amhara, and some parts of Oromia regions. The statistical significance of the primary clusters was also observed in Tigray and Amhara regions.

**Conclusions:**

We found that one-third of Ethiopia’s population is consuming alcohol. Having a cigarette smoking habit, chewing *khat*, high proportion of community literacy, and traditional religion were associated factors for alcohol consumption. Therefore, the federal government of Ethiopia, and ministry of health, and other concerned bodies should work in collaboration to decrease the proportion of people consuming alcohol.

## Introduction

Alcohol consumption is a significant cause of premature mortality and morbidity globally; causing 1.78 million deaths in 2020 [1]. The Global Burden of Disease (GBD) report indicates that about 1.34 billion individuals consumed harmful amounts of alcohol [1]. The consumption of alcohol at any level is linked to health impairments caused by various diseases, such as liver cirrhosis, breast cancer, tuberculosis, and injuries [2–4]. The World Health Organization (WHO) has identified more than 300 diseases and adverse conditions that can result from irresponsible alcohol consumption patterns [5]. Beyond health impairments, alcohol consumption is a significant cause of adverse pregnancy outcomes, such as low birth weight, preterm birth, spontaneous abortion and others, which need urgent interventions [6, 7].

The consumption of alcohol is more prevalent in Sub-Saharan Africa [8]. According to the WHO report, the magnitude of harmful alcohol consumption was found to be 78.9% in Sub-Saharan Africa [5]. Evidence from different countries also revealed that the magnitude of alcohol consumption is as high as 57.9%-76.0% in Nigeria [9, 10], 53.7% in Sri Lanka [11], and 51.4% in Uganda [12]. In Ethiopia, the prevalence of alcohol consumption varied from 16.7% to 72.6% [13, 14]. Evidence from a systematic review and meta-analysis revealed that the magnitude of alcohol consumption could be as high as 44.16% in Ethiopia [15] and 48.23% in Gondar [16]. Evidence from these studies showed that being male [9, 17], being cigarette smoker [18, 19], having lower socioeconomic status (SES) [20–22], and being a follower of a Christian religion (51.6%) [16] were determinant factors for alcohol consumption.

Although numerous investigations have been conducted on alcohol consumption [15, 16, 18], they are limited to specific locations. This could not represent the current alcohol consumption in Ethiopia. Besides, the spatial distribution of alcohol consumption was not well investigated in the previous studies, which could hinder the implementation of effective intervention strategies for alcohol related mortality and morbidity. Therefore this study aimed to answer the following research questions: (i) what is the magnitude of alcohol consumption in Ethiopia? (ii) What are the individual and community level factors which contribute to alcohol consumption in Ethiopia? (iii) Where are the hotspot areas of alcohol consumption in Ethiopia?. The results of this study will provide essential evidence that can inform substance abuse control program planners, policymakers, and healthcare providers. This evidence will be valuable in designing and implementing evidence-based interventions aimed at reducing the burden of alcohol consumption-related mortality and morbidity in Ethiopia and other similar settings.

## Methods and materials

### Study setting and data source

This study used secondary data from the 2016 Ethiopian Demographic Health Survey (EDHS). The EDHS data pool was conducted by the collaboration of EPHI, Federal Ministry of Health (FMoH) and Central Statistical Agency. This survey was conducted in all regions of Ethiopia namely Tigray, Amhara, Oromia, SNNPR (Southern Nations, Nationalities and People Region), Afar, Somali, Gambela, Benishangul Gumuz, Harari, Sidama, Southwest Ethiopia, and two administrative cities (Addis Ababa and Dire Dawa city) [23].

### Sampling procedures and populations

A stratified two-stage cluster sampling method was applied to the 2016 EDHS. The process of stratification involved categorizing the eleven regional states and two city administrations of Ethiopia into urban and rural zones. A total of 84,915 Enumeration Areas (EA) were identified at the initial stage. Of these, 645 EA (202 in urban and 443 in rural areas) were included. At second stage, twenty-eight households per cluster were systematically selected. The enumeration areas that have no latitude and longitude were dropped. Moreover, detailed information about the sampling procedures is available on the DHS website in the 2016 EDHS report [23]. We incorporated the weighted sample of 44,022 study participants.

### Dependent variable

The dependent variable of the current study was alcohol consumption using two EDHS questions. Have you ever taken a drink that contains alcohol? and “during the last 30 days, how many days did you have a drink that contains alcohol?”. Therefore, respondent who consumed alcohol were categorized as “Yes”, coded as “1” otherwise “No”, coded as “0”.

### Independent variables

Individual independent factors, such as the sex of the respondent, age of the household head, religion, marital status, family size, chewing *Khat*, and cigarette smoking were included in this study. Similarly, community-level factors, such as community-level poverty, community-level media exposure, community-level literacy, residence, and region of the study participants were also considered.

### Operational definition

#### Community-level literacy

The proportion of household heads that are educated in primary school and above categories were summed and divided by the total in each cluster. Those household heads scored at or above the mean value of educational level were classified as high community-level literacy, while those scored below the mean score value were classified as low community-level literacy. The mean value was used as the cut-off point for classification because data was normally distributed [24].

#### Community-level media exposure

Was determined using three survey questions: “how often do you have read newspaper or magazine?; how often do you listen to the radio?; and how often do you have watching television? The responses were “not at all”, “at least once a week” and “more than once a week” for each question. The proportions of household heads that are exposed to television, radio, and newspaper were summed and divided by the total media exposure value. Those household heads scored at or above the mean score of the media exposure were classified as high community-level media exposure, while those scored below the mean score value were classified as low community-level media exposure [25].

#### Community-level poverty

Was generated by aggregating the individual characteristics in the clusters (poor, middle and rich). The proportion of household heads classified within the poor wealth index were summed and divided by the total in each cluster. Those households heads scored at or above the mean score value of the wealth index were classified a high poverty level, while those below the mean score value were categorized as low poverty level. The mean value was used as the cut-off point for classification because data was normally distributed.

### Data management and analysis

The extracted data was cleaned, coded, and analyzed using STATA version 14. Sampling weights were also carried out before any analysis. Spatial autocorrelation, interpolation, and SatScan analyses were performed to determine the spatial distribution of alcohol consumption in different regions of Ethiopia. Besides, multilevel logistic regression analysis was employed to identify factors associated with alcohol consumption. Detailed procedures are indicated as follows. In the bivariable analysis, the variables with p-values less than 0.2 were candidate for a multivariable model [26].

### Spatial analysis

ArcGIS version 10.7.1 was used to determine the spatial analysis of alcohol consumption among the 2016 EDHS clusters. The proportions of alcohol consumption cases in each survey cluster were calculated and appended to the latitude and longitude coordinates of the selected enumeration area. Global Moran’s I was used to determine whether alcohol consumption was dispersed, clustered, or randomly distributed in Ethiopia. Moran’s I value close to -1, + 1, and 0 indicate a dispersed, clustered, and random distribution of alcohol consumption, respectively. A p-value of Moran’s I less than 0.05 showed the presence of spatial autocorrelation. Getis-OrdGi* statistics was used to identify the hotspot area of alcohol consumptions and how spatial autocorrelation differs across study locations. The output with a high GI* implies a “hotspot”, whereas a low GI* denotes a “cold spot”. The spatial interpolation technique was also performed to predict alcohol consumption for un-sampled areas from the sampled cluster.

### Spatial SaTscan analysis

Spatial Satscan statistics were performed using Kuldorff’s SaTscan version 10.1 software. Spatial scan statistics were employed using a Bernoulli-based model to identify statistically significant clusters of alcohol consumption among study participants [27]. Household heads who consumed alcohol were considered as cases and those household heads who did not consume alcohol were considered as controls. The result was reported using both table and figure and areas with a high log-likelihood ratio and p-value less than 0.05 were considered to high risk of alcohol consumption. The primary and secondary clusters were identified, assigned p-values, and ranked based on their likelihood ratio test based on the 999 Monte Carlo replications.

### Multi-level analysis

Multilevel analysis was conducted with four models. The first model (I or null model) contains only outcome variable, which help to determine the variability of alcohol consumption in the community. Model II contains only individual-level factors whereas Model III contains only community-level factors. The final model (model IV) contains both individual and community-level factors. In multivariable analysis, the variables p-value less than 0.05 were considered as factors associated with alcohol consumption. The log-likelihood was used for model comparison and fitness and the results showed that Model IV was considered the best–fitted model. Besides, Intra-Class Correlation (ICC), median odds ratio (MOR), and proportional change in variance (PCV) were calculated to measure the variation of alcohol consumption among the household heads or clusters. The ICC is determined using the formula—$ICC=\frac{VA}{VA+\frac{\pi 2}{3}}$, Where, VA represents the area-level variance [28–30]. The $MOR=e^{0.95}\sqrt{VA}$, where VA donates the area level variance [30]. The PCV measures the proportion of total observed individual variation that can be attributed to differences between clusters. The PCV is calculated as—$PCV=\frac{Vnull-VA}{Vnull}$, where, Vnull represents the variance of the initial model, while VA represents the variance of the model with more terms [30].

### Ethical approval

An authorization letter to download EDHS dataset was obtained from DHS portal https://dhsprogram.com/data/dataset_admin/index.cfm) after requesting. The requested data was treated confidentially and was used only for the study purpose. No attempt was done to contact any individual respondent or household included in the survey. The dataset and all methods and data were performed based on Helsinki principles research guidelines.

## Results

### Socio-demographic characteristics

In this analysis, weight samples of 44,022 study participants were included. More than three-fourths (81.8%) of the study participants were male. The majority (88.3%) of the study participants were rural residents. 74.9% of the study participants had no formal education. Similarly, majority of the study participants were aged between 25–64 years (94.2%) followed by ≥ 65 years (4.4%) (Table 1).

**Table 1:** Socio-demographic characteristics of the study participants in Ethiopia, Ethiopian demographic and health survey 2016 (n = 44,022).

Variables	Category	Alcohol consumption		Total weighted frequency (%)
		Yes (14,595)	No (29,427)	
Sex of household head	Male	12,245 (83.9%)	23,764 (80.7%)	36,008 (81.8%)
	Female	2,350 (16.1%)	5,663 (19.3%)	8,014 (18.2%)
Residence	Rural	12,501(85.6%)	26,396 (89.7%)	38,897(88.3%)
	Urban	2,094 (14.3%)	3,032 (10.3%)	5,125 (11.6%)
Family size	≤5	6,319 (43.3%)	9,635 (32.7%)	15,954 (36.2%)
	5	8,276 (56.7%)	19,792 (67.3%)	28,068 (63.8%)
Current marital status	Currently married	13,089 (89.7%)	27,352 (92.9%)	40,441 (91.9%)
	Currently not married	1,506 (10.3%)	2,075 (7.1%)	3,581 (8.1%)
Age of household head (years)	≤24	135 (0.92%)	505 (1.7%)	640 (1.4%)
	25–64	13,859 (94.9%)	27,604 (93.8%)	41,464 (94.2%)
	≥65	601 (4.2%)	1,318 (4.5%)	1,919 (4.4%)
Educational level	No formal education	11,174 (76.6%)	21,833 (74.2%)	33,007 (74.9%)
	Primary and secondary	3,129 (21.4%)	7,238 (24.6%)	10,368 (23.6%)
	Higher	292 (2.0%)	356 (1.2%)	648 (1.5%)
Wealth index	Poor	5,701 (18.5%)	12,919 (43.9%)	18,620 (42.3%)
	Middle	2,965 (20.3%)	6,241 (21.2%)	9,206 (20.9%)
	Rich	5,929 (61.2%0	10,267 (34.9%)	16,196 (36.8%)
Region	Tigray	2,213 (15.2%)	734 (2.5%)	2,947 (6.7%)
	Afar	8 (0.05%)	376 (1.3%)	385 (0.9%)
	Amhara	7,526 (51.6%)	1,926 (6.5%)	9,452 (21.5%)
	Oromia	2,661 (18.2%)	15,340 (52.1%)	18,001 (40.9%)
	Somali	1 (0.006%)	1,702 (5.8%)	1,703 (3.9%)
	Benishangul Gumuz	166 (1.1%)	322 (1.1%)	488 (1.1%)
	SNNPR	1,409 (9.6%)	8,417 (28.6%)	9,826 (22.3%)
	Gambela	26 (0.18%)	73 (0.2%)	99 (0.23%)
	Harari	6 (0.04%)	82 (0.3%)	88 (0.2%)
	Addis Ababa	561(3.8%)	289 (0.9%)	850 (1.9%)
	Dire Dawa	18 (0.12%)	166 (0.5%)	184 (0.42%)
Community-level media exposure	Low	10,089 (69.2%)	21,222 (72.1%)	31,311 (71.1%)
	High	4,506 (30.8%)	8,205 (27.9%)	12,711 (28.9%)
Chewing *khat*	Yes	457 (3.1%)	6,584 (22.4%)	7,041(16%)
	No	14,138 (96.9%)	22,843 (77.6%)	36,981 (84%)
Smoking cigarette	Yes	159 (1.1%)	220 (0.7%)	379 (0.9%)
	No	14,436 (98.9%)	29,207 (99.3%)	43,643 (99.1%)
Religion	Orthodox	13,402 (91.8%)	3,110 (10.6%)	16,512 (37.5%)
	Catholic	37 (0.25%)	269 (0.9%)	306 (0.7%)
	Muslim	376 (2.6%)	15,878 (53.9%)	16,254 (36.9%)
	Protestant	605 (4.1%)	9,588 (32.6%)	10,193 (23.1%)
	Other*	175 (1.1%)	582 (2.0%)	757 (1.8%)

Other*: traditional religion

### Spatial autocorrelation of alcohol consumption

**Fig 1:**
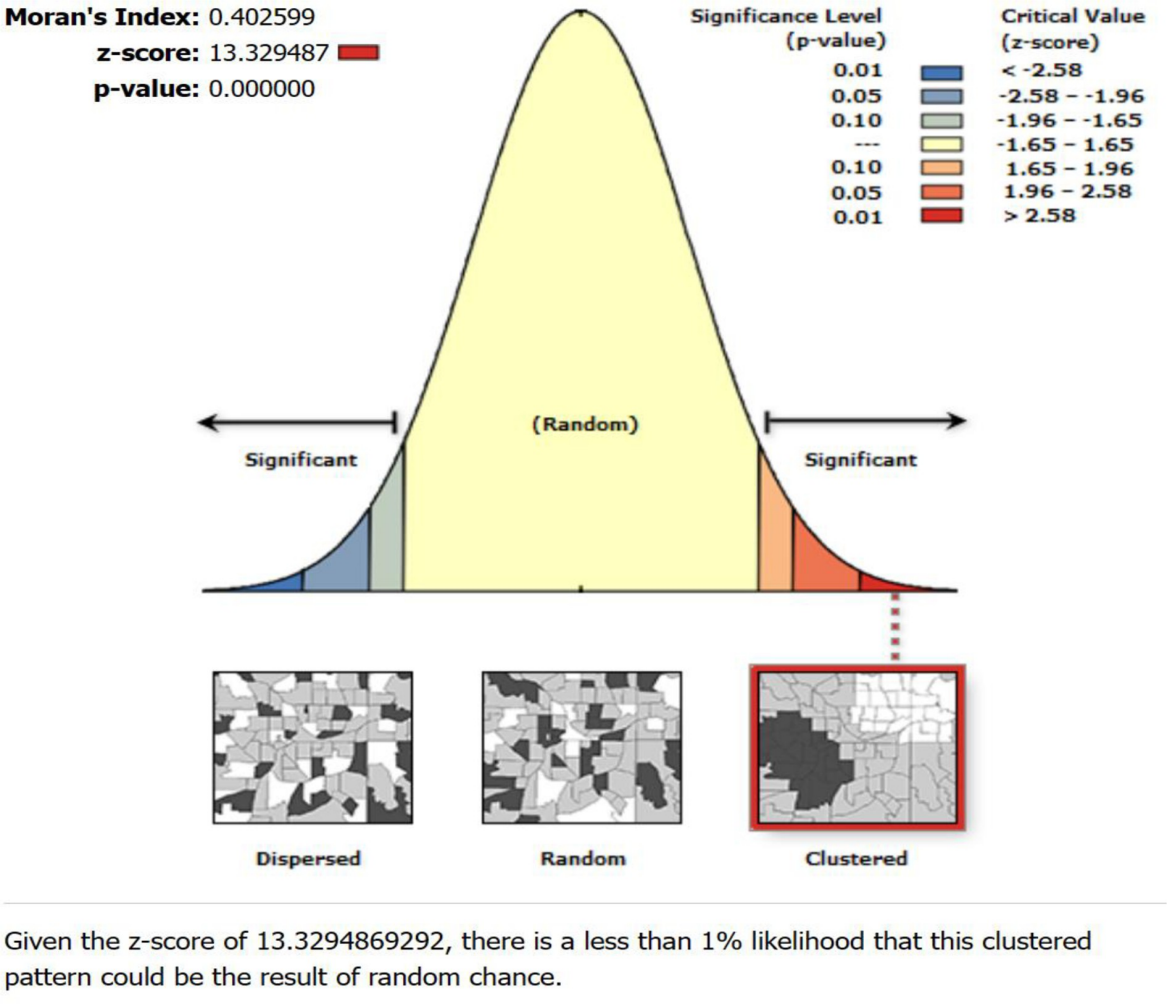
Spatial autocorrelation of alcohol consumption in Ethiopia using the 2016 Ethiopian demographic and health survey data.

The prevalence of alcohol consumption spatially varied across regions of Ethiopia, as shown in the cluster pattern. We found a Global Moran’s I value of 0.4 with statistically significant variation (p < 0.001). The Z-score value was found to be 13.3 which indicates that there was less than a 1% probability that this clustered pattern has been the result of a random chance (Fig 1).

### Prevalence and spatial distribution of alcohol consumption in Ethiopia

**Fig 2:**
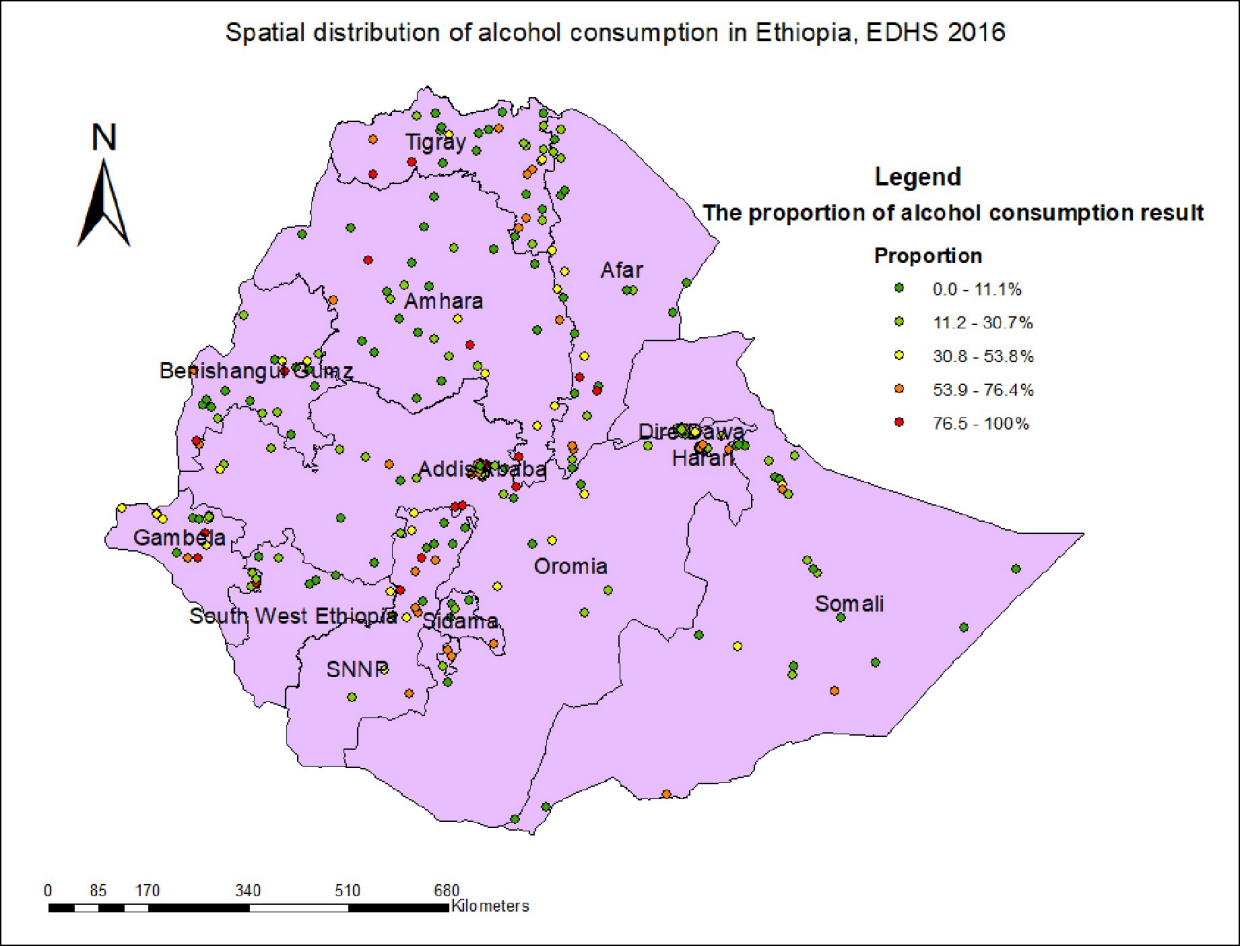
Spatial distribution of alcohol consumption in Ethiopia, using EDHS 2016 dataset.

The prevalence of alcohol consumption in Ethiopia was found to be 33.15% (95%CI: 32.5–33.8). As shown in Fig 2, the spatial distribution of alcohol consumption varied across the region. The spatial distribution of alcohol consumption has been observed in the Sidama, SNNP, Amhara, Tigray, Gambela, Benishangul gumuz, and some parts of the Oromia region (Fig 2).

### Prevalence of alcohol consumption within past 30 days

Out of lifetime alcohol use (14,595; 33.15%), 13,521 (30.7%) of the study participants were drink the alcohol within past 30 days. Out of 30.7% of drink alcohol within past 30 days, majority of study participants were drink the alcohol 1–10 days (58.3%) followed by 11–20 days (Table 2).

**Table 2:** Prevalence of alcohol consumption within the past 30 days in Ethiopia using EDHS 2016 dataset (n = 14,595).

During the last 30 days, how many days did you have a drink the alcohol?	**Category (days)**	**Weighted frequency**	**Percentage (%)**
	1–10 days	7,882	58.3
	11–20	3,542	26.2
	21–30	2,095	15.5
	Total	13,521	100%

### Hot spot analysis of alcohol consumption

**Fig 3:**
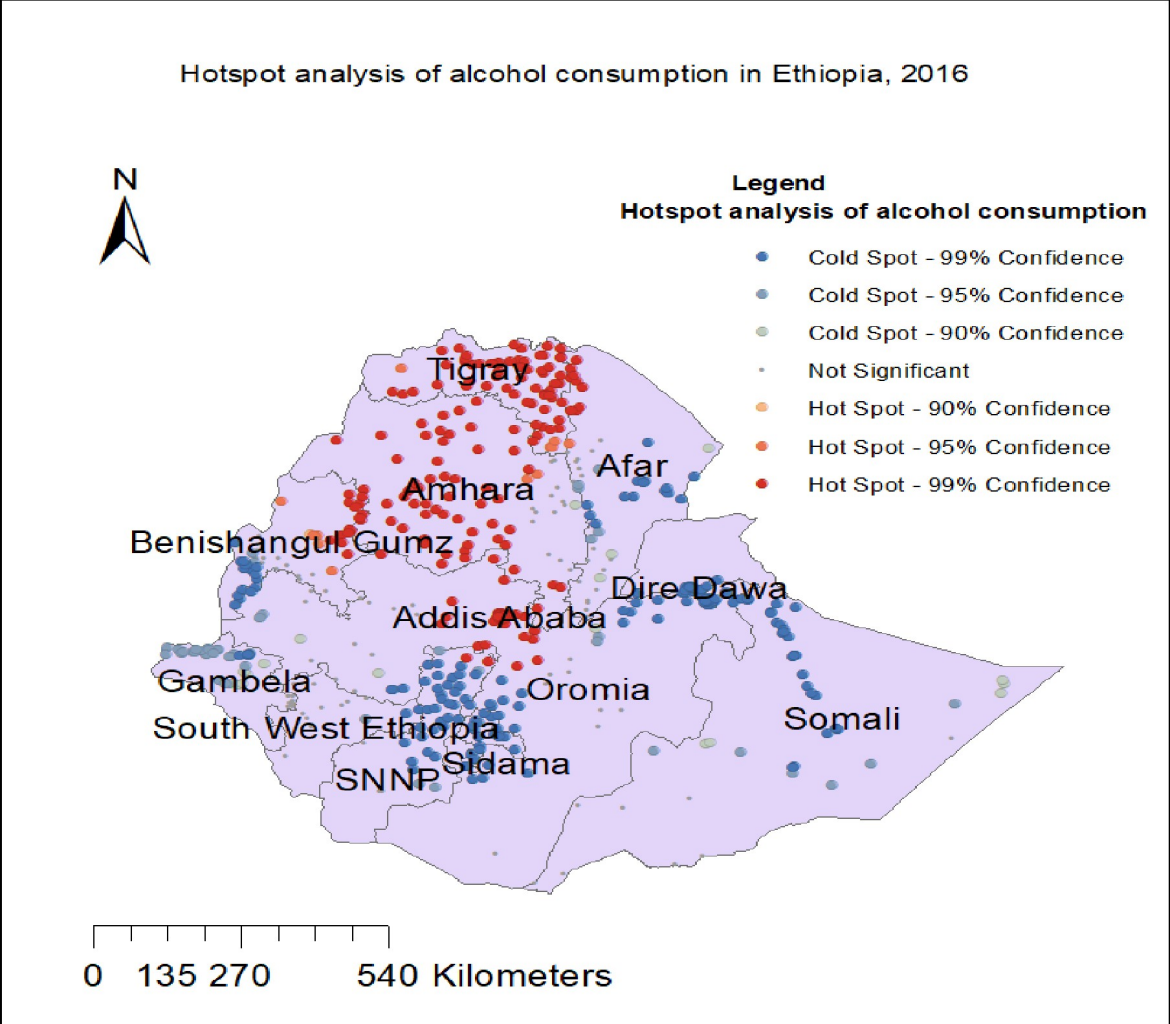
The hotspot analysis of alcohol consumption in Ethiopia, using the EDHS 2016 dataset.

Significant high proportion (hotspot) areas of alcohol consumption were indicated in red color and they were observed in Amhara, Tigray, Oromia, Benishangul Gumuz region, and Addis Ababa. The low proportion (cold spot) areas of alcohol consumption were observed in Somali, Sidama, SNNP, Gambela, Dire Dawa, and Harari regions of Ethiopia as indicated in blue color (Fig 3).

### Spatial interpolation of alcohol consumption

**Fig 4:**
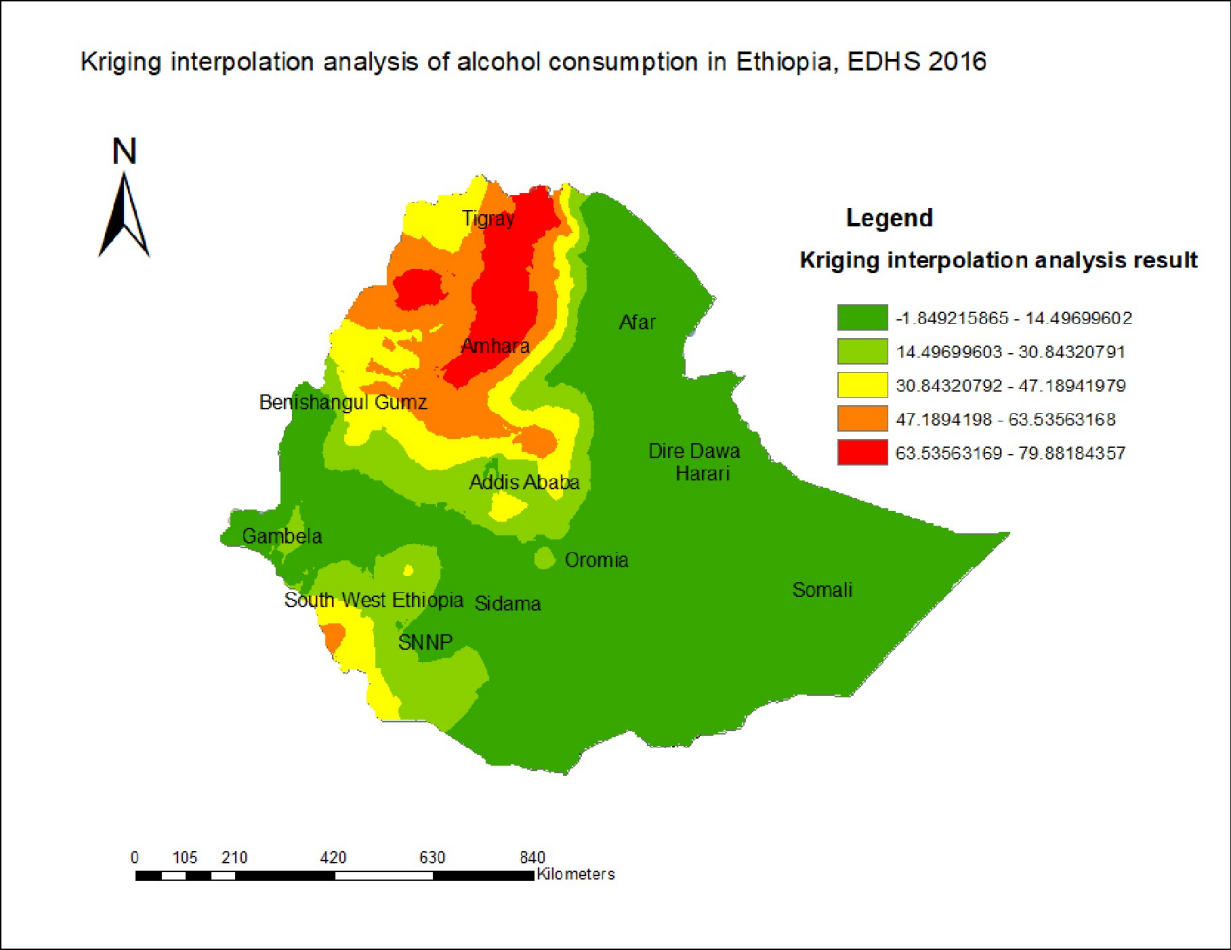
Spatial interpolation of alcohol consumption in Ethiopia, using the EDHS 2016 dataset.

According to ordinary Gaussian Kriging interpolation results, the predicted high proportion area for alcohol consumption was extremely high ranging from 63.5% - 79.8% in Amhara and Tigray regions, which was presented in red color. On the other hand, the lower predicted alcohol consumption has been observed in Somali, Afar, Harari, Dire Dawa, Sidama, and some parts of Oromia and SNNPR (Fig 4).

### Spatial scan statistical analysis

**Fig 5:**
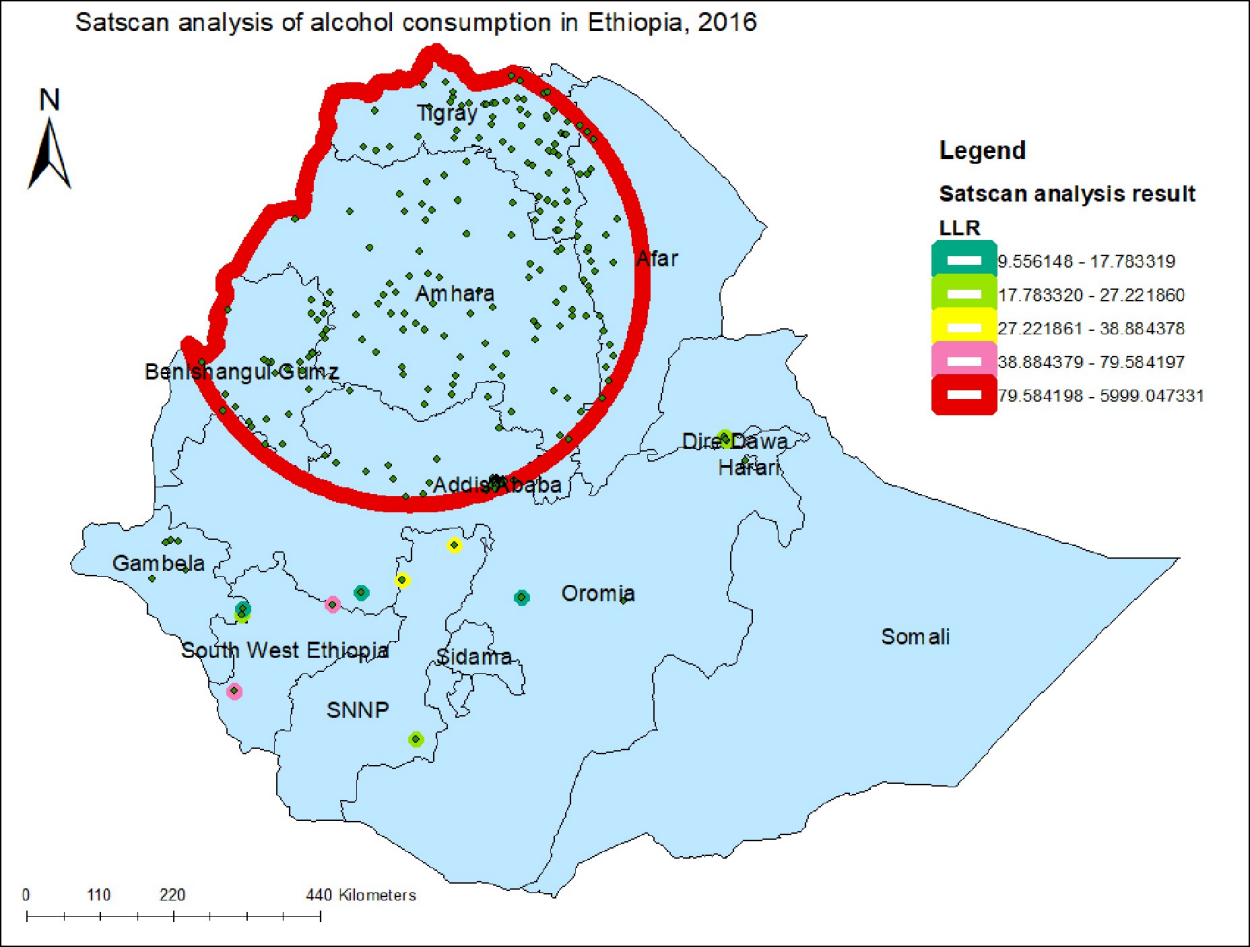
SatScan analysis of alcohol consumption in Ethiopia using the EDHS 2016 dataset.

A total of 257 clusters were statistically significant; of which 252 were most likely (primary) and 5 secondary clusters. The primary cluster spatial window (red color) was in Tigray and Amhara regions that were centered at 11.793026N, 37.584665E with 342.42km radius, a Relative Risk (RR) of 5.81 and Log-Likelihood Ratio of 5999.04, at p-value <0.001. It indicates that the household head living in the window red color had a 5.81 times higher risk of drinking alcohol than the household head living outside the window (Fig 5). Similarly, the secondary statistically significant clusters were found in Oromia and southwest Ethiopia regions that were centered at 9.584129N, 41.866112E with 0.46km radius, a relative risk of 2.38, and log-likelihood ratio of 27.22 at p-value < 0.001; which revealed that the household head living in the second window had 2.38 times higher risk of consuming alcohol than household head living outside of the spatial window (Table 3).

**Table 3:** SatScan analysis result of alcohol consumption in Ethiopia, 2016.

Type of Cluster	Enumeration area (EA)	Coordinate/ radius	Populations	Case	RR	LLR	p-value
Primary	158, 512, 169, 73, 163, 132, 431, 456, 167, 516, 382, 403, 292, 429, 24, 327, 361, 640, 120, 627, 152, 38, 199, 312, 109, 259, 3, 638, 602, 545, 279, 628, 375, 541, 206, 322, 474, 515, 415, 548, 176, 498, 615, 386, 80, 66, 482, 531, 229, 591, 401, 10, 460, 533, 246, 542, 350, 52, 559, 494, 425, 478, 218, 354, 296, 612, 267, 616, 300, 36, 504, 617, 258, 150, 510, 136, 188, 410, 200, 392, 97, 184, 455, 183, 496, 351, 340, 551, 143, 18, 442, 449, 137, 575, 538, 611, 579, 364, 424, 244, 345, 35, 572, 249, 253, 189, 160, 583, 128, 156, 488, 332, 423, 79, 571, 191, 254, 344, 181, 241, 636, 98, 310, 256, 389, 320, 584, 255, 430, 237, 517, 550, 94, 355, 597, 400, 605, 384, 528, 399, 368, 590, 81, 268, 220, 421, 511, 544, 161, 55, 637, 78, 481, 604, 280, 294, 84, 348, 88, 130, 234, 623, 45, 129, 99, 547, 172, 485, 226, 461, 298, 70, 599, 620, 124, 65, 276, 569, 295, 621, 201, 274, 341, 196, 23, 463, 112, 532, 144, 11, 91, 349, 339, 369, 464, 626, 118, 107, 170, 31, 153, 108, 100, 283, 457, 305, 582, 635, 247, 15, 414, 287, 314, 487, 195, 639, 59, 645, 608, 145, 159, 509, 428, 560, 90, 89, 479, 19, 155, 293, 127, 402, 302, 110, 102, 264, 598, 225, 624, 211, 330, 61, 235, 585, 451, 539, 147, 303, 335, 404, 261, 475	11.793026N, 37.584665E/ 342.42km	15139	9471	5.81	5999.04	<0.0001
Secondary	471, 519, 535	9.584129N, 41.866112E/0.46km	73	53	2.38	27.22	<0.0001
Secondary I	101, 140	9.620244N, 41.840029E/0.45km	66	47	2.33	22.99	<0.0001

Hint: RR- Relative risk; LLR- Log-Likelihood Ratio

### Multilevel logistic regression analysis

#### Random effect model analysis

The log odds of alcohol consumption were statistically significant among the community. According to the intra-class correlation (ICC) in the null model, 86% of the variation in alcohol consumption was attributed to community-level factors. The null model also had the highest median odds ratio (MOR) value of 11.74, indicating that if we randomly select study participants from two different clusters, the study participants in the cluster with a high risk of alcohol consumption had 11.74 times higher odds of alcohol consumption than study participants in the cluster with a lower risk of alcohol consumption. Similarly, the percentage of PCV increases from 73.3% in Model I to 84.7% in Model III, which indicates that the third model was best in explaining the variations of alcohol consumption (Table 4).

**Table 4:** Random effect (community level-clustering) and model selection for alcohol consumption in Ethiopia, 2016.

Random effect				
	Null-model	Model I	Model II	Model III
VA	20.5841	5.485	6.16977	3.153621
ICC	0.86	0.625	0.652	0.489
MOR	11.74	6.06	6.42	4.59
LLR	-12763.149	-9566.2542	-11835.009	-9205.0429
PCV	Reference	0.733	0.70	0.847

#### Factors associated with alcohol consumption

Based on the final model of multilevel multivariable logistic regression, religion, region, community level literacy, and behavioral factors, such as cigarette smoking and chewing *Khat*, were factors associated with alcohol consumption. The odds of alcohol consumption were thirteen times higher among traditional religion followers as compared to protestant religion followers (AOR = 13.7; 95%CI: 2.68–70.3). Household heads in the Amhara region were three times more likely to drink alcohol as compared to these in other regions (AOR = 3.56; 95%CI: 1.85–6.8). The odds of alcohol consumption were almost two times higher among these with low literacy as compared to participants with high literacy (AOR = 1.84; 95%CI: 1.1–3.18). Similarly, the odds of alcohol consumption were fifteen times higher among cigarette smokers as compared to their counterparts (OR = 15.82; 95%CI: 4.31–58.1). Those study participants who chew *Khat* were also three times more likely to consume alcohol as compared to those who did not chew *Khat* (AOR = 2.98; 95%CI: 1.22–7.27) (Table 3). On the other hands, the odds of alcohol consumption were 89% times lower among muslim religion followers as compared to their counterparts (AOR = 0.11; 95%CI: 0.04–0.36) (Table 5).

**Table 5:** Multivariable multilevel logistic regression analysis of alcohol consumption in Ethiopia, 2016.

Variable name	Category	Null model	Model I [AOR 95%CI]	Model II [AOR 95%CI]	Model III [AOR 95%CI]
Sex of household head	Male		1.26 (0.81–1.98)		1.24 (0.79–1.49)
	Female		1		1
Age of household head (years)	≥65		1.43 (0.58–3.55)		1.43 (0.58–3.6)
	25–64		1.31 (0.63–2.72)		1.29 (0.62–2.72)
	15–24		1		1
Family size	>5		0.88 (0.63–1.21)		0.89(0.64–1.22)
	≤5		1		1
Marital status	Not currently married		0.81 (0.48–1.35)		0.78 (0.47–1.31)
	Currently married		1		1
Cigarette smoking	Yes		16.67 (4.3–64.62)*		15.82 (4.31–58.1)*
	No		1		1
Chewing Khat	Yes		3.1 (1.25–7.57)*		2.98 (1.22–7.27)*
	No		1		1
Religion	Orthodox		29.6(16.37–53.53)*		25.86 (14.7–45.5)*
	Catholic		4.2(0.83–20.89)		4.1(0.89–18.5)
	Muslim		0.09 (0.03–0.29)*		0.11 (0.04–0.36)*
	Others*		16.67 (2.68–80.37)*		13.7 (2.68–70.3)*
	Protestant		1		1
Region	Afar			0.01 (0.001–0.025)	0.04 (0.015–0.13)
	Amhara			2.0 (0.91–4.42)	3.56 (1.85–6.8)*
	Oromia			0.005 (0.002–0.01)	0.07 (0.03–0.15)
	Somali			0.001 (0.0001–0.006)	0.003 (0.0004–0.018)
	Benishangulumuz			0.06 (0.03–0.13)	0.7 (0.31–1.42)
	SNNPR			0.005 (0.002–0.11)	0.06 (0.03–0.13)
	Gambela			0.03 (0.02–0.07)	0.4 (0.18–0.8)
	Harari			0.003(0.001–0.006)	0.1 (0.03–0.18)
	Addis Ababa			0.21 (0.1–0.4)	1.2 (0.63–2.4)
	Dire Dawa			0.005 (0.002–0.012)	0.1 (0.04–0.23)
Community literacy level	Low			1.8 (1.03–3.27)*	1.84 (1.1–3.18)*
	High			1	1
Community media exposure	No media exposure			2.24 (1.19–4.2)*	1.66 (0.97–2.84)
	Have media exposure			1	1
Residence	Rural			0.6 (0.27–1.32)	1.1 (0.54–2.11)
	Urban			1	1
Community poverty	Low			0.6 (0.3–1.22)	0.95 (0.53–1.69)
	High			1	1

Hint: 1: reference; AOR: adjusted odds ratio; others religion

*: traditional

## Discussion

Alcohol consumption continues to be a significant cause of premature mortality and morbidity in Ethiopia. This study aimed to determine the spatial distribution and factors associated with alcohol consumption using the EDHS 2016 dataset. The prevalence of alcohol consumption was found to be 33.15%. The current finding was lower than the previous study findings in different regions of the world, such as Ethiopia (72.6%) [13, 14], New Zealand (95%) [31], India (49.7%) [32], Nigeria (57.9%) [9], Sri Lanka (53.7%) [11], Uganda (51.4%) [12], and Nigeria (76.0%) [10], and Ghana (39.5%) [33]. In the contrary, the current finding was greater than these reported in Kenya (10.8%) [34], Ambo, Ethiopia (27%) [35], and systematic review and meta-analysis finding in Ethiopia (23.86%) [15]. But, it is in agreement with the global status report on alcohol and health in Sub-Saharan Africa (32.7%) [5, 36]. This difference might be due to the difference of the study population and sample size. Majorities of the previous focused on the students only, which could vary the prevalence of alcohol consumption. In this study, the spatial distribution of alcohol consumption was high among Amhara, Tigray, and some parts of the Oromia region. This might be because majority of the community in these regions are more agrarian where local alcoholic beverages are highly produced, which could increase alcohol consumption in the area [37].

The odds of alcohol consumption were three times higher among Orthodox religion followers as compared to protestants. Similarly, the odds of alcohol consumption were thirteen times higher among traditional religion followers as compared to protestants. On the other hand, Muslim religion followers have even 89% lower odds of alcohol consumption compared to protestants. The finding is in agreement with studies conducted in Nigeria [9] and Brazil [17]. This consistency might be due to the fact that alcohol consumption is culturally and socially accepted by Orthodox religious followers in Ethiopia.

The odds of alcohol consumption were almost two times higher among respondents living in low proportion of community literacy as compared to high proportion of community literacy. The association might be due to low level of awareness about the health effects of alcohol consumption are high among household heads living in low community’s literacy. In other words, household heads living with a high proportion of community literacy might assist household heads in obtaining better knowledge about the health effects of alcohol consumption that could decrease alcohol consumption.

Once more, study participants that cigarette smoking habits had fifteen times higher odds of alcohol consumption than those who had no cigarette smoking habit. This finding is supported by other reports [8, 17, 18]. Smoking cigarettes can significantly influence alcohol consumption in the communities. The co-occurrence of these behaviors is common due to shared addictive qualities. Nicotine in cigarettes can enhance the pleasurable effects of alcohol, leading to increased alcohol consumption.

Similarly, study participants that chew *Khat* had three times higher odds of alcohol consumption as compared to their counterparts. Chewing *khat* can have a notable impact on the communities’ alcohol consumption. Social settings where chewing *khat* occurs can normalize alcohol consumption. The communities may also use alcohol to counteract the effects of *khat*. These findings highlight the complexity of substance use behaviors among communities, necessitating a comprehensive approach to address multiple risk factors simultaneously.

### Strengths and limitations of the study

This study was conducted in all regions of Ethiopia with a large sample size. Multilevel logistic regression analysis and spatial analysis were used to determine the spatial variation of alcohol consumption and its associated factors, which could identify the specific areas for intervention. However, as EDHS data was collected using a cross-sectional study design, it could limit the causality of predictors on alcohol consumption. The quantity of alcohol consumption was not determined in this study since the data did not contain this information. Additionally, the prevalence of alcohol consumption was also not determined in the current study.

## Conclusions

The prevalence of lifetime alcohol consumption was found to be high in Ethiopia. Religion, region, and community literacy level were factors associated with alcohol consumption. Behavioral factors, such as chewing *khat* and cigarette smoking, were also identified as significant predictors of alcohol consumption. Spatial analysis showed that there was a significant clustering of alcohol consumption in Amhara, Tigray, and some parts of the Oromia region. Therefore, the ministry of health, religious leaders, and other concerned bodies should provide compressive public health interventions that target hot spot areas. Substance abuse control law enforcement should be strengthened in Ethiopia.
